# Poly[tetra­deca­aqua­tetra­kis­(μ_3_-1*H*-imidazole-4,5-dicarboxyl­ato)tetra-μ_3_-sulfato-cobalt(II)hexa­gadolinium(III)]

**DOI:** 10.1107/S1600536811026821

**Published:** 2011-07-09

**Authors:** Li-Cai Zhu

**Affiliations:** a School of Chemistry and Environment, South China Normal University, Guangzhou 510631, People’s Republic of China

## Abstract

The asymmetric unit of the title compound, [CoGd_6_(C_5_H_2_N_2_O_4_)_4_(SO_4_)_6_(H_2_O)_14_]_*n*_, contains a Co^II^ ion (site symmetry 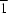), three Gd^III^ ions, two imidazole-4,5-dicarboxyl­ate ligands, three SO_4_^2−^ anions, and seven coordinated water mol­ecules. The Co^II^ ion is six-coordinated by two O atoms from water mol­ecules, two O atoms and two N atoms from two imidazole-4,5-dicarboxyl­ate ligands, giving a slightly distorted octa­hedral geometry. The Gd^III^ ions exhibit three types of coordination environments. One Gd ion is eight-coordinated in a bicapped trigonal–prismatic geometry by four O atoms from two imidazole-4,5-dicarboxyl­ate ligands, two O atoms from two SO_4_^2−^ anions and two coordinated water mol­ecules. The other Gd ions are nine-coordinated in a tricapped trigonal–prismatic geometry; one of these Gd ions is bonded to four O atoms from two imidazole-4,5-dicarboxyl­ate ligands, three O atoms from three SO_4_^2−^ anions and two water O atoms and the other Gd ion is coordinated by one O atom and one N atom from one imidazole-4, 5-dicarboxyl­ate ligand, five O atoms from three SO_4_^2−^ anions as well as two coordinated water mol­ecules. These metal coordination units are connected by bridging imidazole-4,5-dicarboxyl­ate and sulfate ligands, generating a three-dimensional network. The crystal structure is further stabilized by N—H⋯O, O—H⋯O, and C—H⋯O hydrogen-bonding inter­actions between water mol­ecules, SO_4_^2−^ anions, and imidazole-4,5-dicarboxyl­ate ligands.

## Related literature

For applications and crystal structures of related compounds, see: Cheng *et al.* (2006 ▶); Kuang *et al.* (2007 ▶); Sun & Yang (2007 ▶); Zhu *et al.* (2010 ▶).
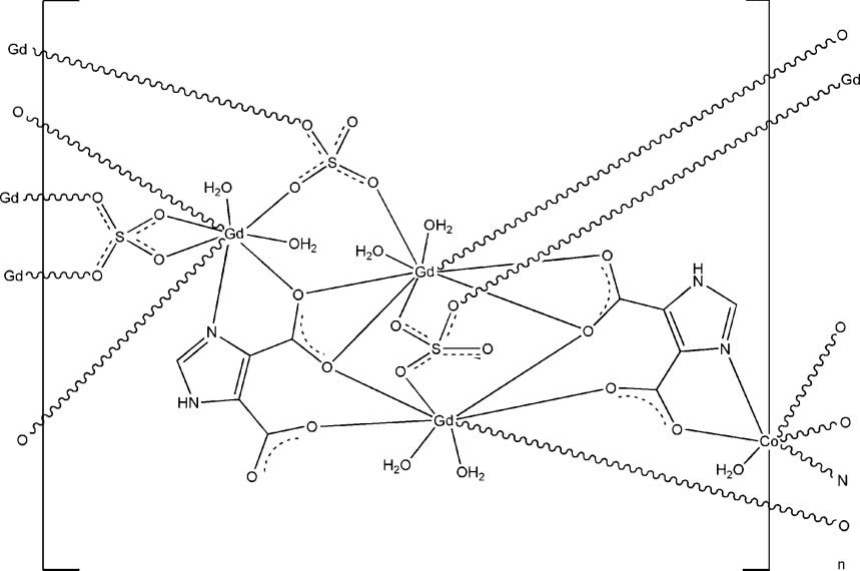


## Experimental

### 

#### Crystal data

[CoGd_6_(C_5_H_2_N_2_O_4_)_4_(SO_4_)_6_(H_2_O)_14_]
*M* *_r_* = 2447.42
Triclinic, 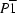

*a* = 6.6246 (4) Å
*b* = 9.4895 (6) Å
*c* = 21.5242 (14) Å
α = 97.037 (1)°
β = 94.365 (1)°
γ = 98.158 (1)°
*V* = 1323.28 (14) Å^3^
*Z* = 1
Mo *K*α radiation
μ = 8.10 mm^−1^
*T* = 296 K
0.20 × 0.18 × 0.15 mm

#### Data collection

Bruker APEXII area-detector diffractometer
Absorption correction: multi-scan (*SADABS*; Sheldrick, 1996 ▶) *T*_min_ = 0.216, *T*_max_ = 0.297
6864 measured reflections
4682 independent reflections
4123 reflections with *I* > 2σ(*I*)
*R*_int_ = 0.019

#### Refinement

*R*[*F* ^2^ > 2σ(*F* ^2^)] = 0.026
*wR*(*F* ^2^) = 0.059
*S* = 1.04
4682 reflections
478 parameters
23 restraints
H atoms treated by a mixture of independent and constrained refinement
Δρ_max_ = 0.95 e Å^−3^
Δρ_min_ = −0.89 e Å^−3^

### 

Data collection: *APEX2* (Bruker, 2004 ▶); cell refinement: *SAINT* (Bruker, 2004 ▶); data reduction: *SAINT*; program(s) used to solve structure: *SHELXS97* (Sheldrick, 2008 ▶); program(s) used to refine structure: *SHELXL97* (Sheldrick, 2008 ▶); molecular graphics: *XP* in *SHELXTL* (Sheldrick, 2008 ▶); software used to prepare material for publication: *SHELXL97*.

## Supplementary Material

Crystal structure: contains datablock(s) I, global. DOI: 10.1107/S1600536811026821/pv2424sup1.cif

Structure factors: contains datablock(s) I. DOI: 10.1107/S1600536811026821/pv2424Isup2.hkl

Additional supplementary materials:  crystallographic information; 3D view; checkCIF report

## Figures and Tables

**Table 1:** Hydrogen-bond geometry (Å, °)

*D*—H⋯*A*	*D*—H	H⋯*A*	*D*⋯*A*	*D*—H⋯*A*
N4—H1⋯O7^i^	0.86 (3)	1.93 (4)	2.770 (6)	166 (5)
N2—H2⋯O15^ii^	0.87 (4)	2.03 (4)	2.865 (7)	161 (4)
O1*W*—H2*W*⋯O17^iii^	0.81 (4)	2.07 (4)	2.785 (6)	148 (6)
O2*W*—H4*W*⋯O3*W*	0.83 (3)	2.04 (4)	2.817 (6)	158 (4)
O3*W*—H5*W*⋯O4^iv^	0.82 (4)	1.93 (4)	2.720 (6)	159 (4)
O3*W*—H6*W*⋯O3^v^	0.84 (4)	1.96 (4)	2.779 (6)	167 (6)
O4*W*—H7*W*⋯O13^vi^	0.81 (4)	2.19 (5)	2.888 (6)	144 (4)
O4*W*—H7*W*⋯O14^vi^	0.81 (4)	2.44 (4)	3.175 (6)	150 (5)
O4*W*—H8*W*⋯O8^vi^	0.82 (5)	2.54 (5)	3.217 (6)	141 (4)
O4*W*—H8*W*⋯O2^v^	0.82 (5)	2.54 (5)	3.028 (6)	120 (5)
O5*W*—H9*W*⋯O11^vii^	0.82 (4)	1.83 (4)	2.645 (6)	177 (6)
O5*W*—H10*W*⋯O11^viii^	0.81 (4)	2.07 (5)	2.862 (6)	167 (5)
O5*W*—H10*W*⋯O12^viii^	0.81 (4)	2.44 (4)	3.056 (6)	134 (5)
O6*W*—H12*W*⋯O5*W*^viii^	0.82 (4)	1.98 (3)	2.779 (7)	164 (6)
O7*W*—H13*W*⋯O20^ix^	0.81 (4)	1.99 (5)	2.768 (6)	161 (4)
O7*W*—H14*W*⋯O6*W*^ix^	0.81 (5)	2.31 (5)	3.090 (7)	165 (5)
C3—H3⋯O2^x^	0.93	2.46	3.336 (7)	157

Symmetry codes: (i) 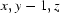; (ii) 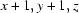; (iii) 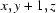; (iv) 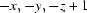; (v) 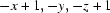; (vi) 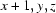; (vii) 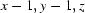; (viii) 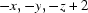; (ix) 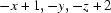; (x) 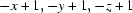.

**Fig. 1.**
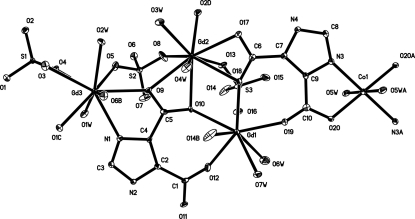
The molecular structure showing the atomic-numbering scheme and displacement ellipsoids drawn at the 30% probability level. Hydrogen atoms were omitted for clarity. Symmetry codes: (A) -x, -1 - y, 2 - z; (B) 1 + x, y, z; (C) 1 - x, 1 - y, 1 - z; (D) -x, -y, 1 - z.

**Fig. 2.**
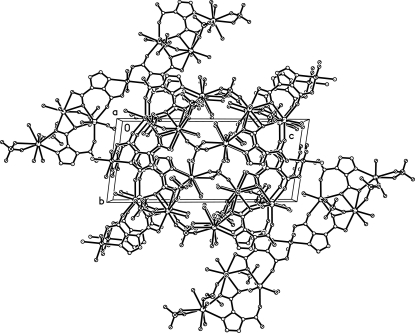
A view of the three-dimensional structure of the title compound. Hydrogen atoms were omitted for clarity.

## supplementary crystallographic information

### Comment

In the past few years, lanthanide-transition metal heterometallic complexs with bridging multifunctionnal organic ligands gained increasing interest, not only because of their impressive topological structures, but also due to their versatile applications in ion exchange, magnetism, bimetallic catalysis and luminescent probe (Cheng *et al.*, 2006; Kuang *et al.*, 2007; Sun *et al.*, 2007; Zhu *et al.*, 2010). As an extension of this research, the structure of the title compound, a new heterometallic coordination polymer, (I), has been determined which is presented in this artcle.

The asymmetric unit of the title compound (Fig. 1), contains a half Co^II^ ion, three Gd^III^ ions, two imidazole-4, 5-dicarboxylate ligands, three SO_4_^2-^ anions, and seven coordinated water molecules. The Co^II^ ion lie in the inversion center and is six-coordinated with two O atoms from two coordinated water molecules, two O atoms and two N atoms from two imidazole-4, 5-dicarboxylate ligands, to give a slightly distorted octahedral geometry. The Gd^III^ ions exhibit three types of coordination environment. Gd(1) ion is eight-coordinated in a bicapped trigonal prismatic coordination geometry by four O atoms from two imidazole-4,5-dicarboxylate ligands, two O atoms from two SO_4_^2-^ anions and two coordinated water molecules. Both Gd(2) ion and Gd(3) ion are nine-coordinated in a tricapped trigonal prismatic coordination geometry. Gd(2) ion is bonded to four O atoms from two imidazole-4,5-dicarboxylate ligands, three O atoms from three SO_4_^2-^ anions and two water O atoms; Gd(3) ion is coordinated by one O atom and one N atom from one imidazole-4,5-dicarboxylate ligand, five O atoms from three SO_4_^2-^ anions as well as two coordinated water molecules. These metal coordination units are connected by bridging imidazole-4,5-dicarboxylate and sulfate ligands, generating a three- dimensional network (Fig. 2). The crystal structure is further stabilized by N—H···O, O—H···O, and C—H···O hydrogen-bonding interactions between water molecules, SO_4_^2-^ anions, and imidazole-4,5-dicarboxylate ligands(Table 1).

### Experimental

A mixture of CoSO_4_.7H_2_O (0.141 g, 0.5 mmol), Gd_2_O_3_ (0.09 g, 0.25 mmol), imidazole-4,5-dicarboxylic acid (0.156 g, 1 mmol), and H_2_O (10 ml) was sealed in a 20 ml Teflon-lined reaction vessel at 443 K for 5 days then slowly cooled to room temperature. The product was collected by filtration, washed with water and air-dried. Red block crystals suitable for X-ray analysis were obtained.

### Refinement

H atoms bonded to C atoms were positioned geometrically and refined as riding, with C—H = 0.93 Å and *U*_iso_(H) = 1.2 *U*_eq_(C). H atoms bonded to N atoms and H atoms of water molecules were found from difference Fourier maps and refined isotropically with a restraint of N—H = 0.87 Å, O—H = 0.82 Å and *U*_iso_(H) = 1.5 *U*_eq_(N, O).

### Crystal data

**Table:**

[CoGd_6_(C_5_H_2_N_2_O_4_)_4_(SO_4_)_6_(H_2_O)_14_]	*Z* = 1
*M**_r_* = 2447.42	*F*(000) = 1151
Triclinic, *P*1	*D*_x_ = 3.071 Mg m^−^^3^
Hall symbol: -P 1	Mo *K*α radiation, λ = 0.71073 Å
*a* = 6.6246 (4) Å	Cell parameters from 3440 reflections
*b* = 9.4895 (6) Å	θ = 2.5–28.0°
*c* = 21.5242 (14) Å	µ = 8.10 mm^−^^1^
α = 97.037 (1)°	*T* = 296 K
β = 94.365 (1)°	Block, red
γ = 98.158 (1)°	0.20 × 0.18 × 0.15 mm
*V* = 1323.28 (14) Å^3^	

### Data collection

**Table:**

Bruker APEXII area-detector diffractometer	4682 independent reflections
Radiation source: fine-focus sealed tube	4123 reflections with *I* > 2σ(*I*)
graphite	*R*_int_ = 0.019
φ and ω scans	θ_max_ = 25.2°, θ_min_ = 1.9°
Absorption correction: multi-scan (*SADABS*; Sheldrick, 1996)	*h* = −7→7
*T*_min_ = 0.216, *T*_max_ = 0.297	*k* = −10→11
6864 measured reflections	*l* = −25→24

### Refinement

**Table:**

Refinement on *F*^2^	Primary atom site location: structure-invariant direct methods
Least-squares matrix: full	Secondary atom site location: difference Fourier map
*R*[*F*^2^ > 2σ(*F*^2^)] = 0.026	Hydrogen site location: inferred from neighbouring sites
*wR*(*F*^2^) = 0.059	H atoms treated by a mixture of independent and constrained refinement
*S* = 1.04	*w* = 1/[σ^2^(*F*_o_^2^) + (0.0266*P*)^2^ + 0.2633*P*] where *P* = (*F*_o_^2^ + 2*F*_c_^2^)/3
4682 reflections	(Δ/σ)_max_ = 0.002
478 parameters	Δρ_max_ = 0.95 e Å^−^^3^
23 restraints	Δρ_min_ = −0.89 e Å^−^^3^

### Special details

**Table:**

Geometry. All e.s.d.'s (except the e.s.d. in the dihedral angle between two l.s. planes) are estimated using the full covariance matrix. The cell e.s.d.'s are taken into account individually in the estimation of e.s.d.'s in distances, angles and torsion angles; correlations between e.s.d.'s in cell parameters are only used when they are defined by crystal symmetry. An approximate (isotropic) treatment of cell e.s.d.'s is used for estimating e.s.d.'s involving l.s. planes.
Refinement. Refinement of *F*^2^ against ALL reflections. The weighted *R*-factor *wR* and goodness of fit *S* are based on *F*^2^, conventional *R*-factors *R* are based on *F*, with *F* set to zero for negative *F*^2^. The threshold expression of *F*^2^ > σ(*F*^2^) is used only for calculating *R*-factors(gt) *etc*. and is not relevant to the choice of reflections for refinement. *R*-factors based on *F*^2^ are statistically about twice as large as those based on *F*, and *R*- factors based on ALL data will be even larger.

### Fractional atomic coordinates and isotropic or equivalent isotropic displacement parameters (Å^2^)

**Table:**

	*x*	*y*	*z*	*U*_iso_*/*U*_eq_	
Gd1	0.32032 (4)	0.01920 (3)	0.860942 (13)	0.01532 (8)	
Gd2	0.06244 (4)	−0.11452 (3)	0.677435 (12)	0.01124 (8)	
Gd3	0.34777 (4)	0.29254 (3)	0.577800 (12)	0.01114 (8)	
Co1	0.0000	−0.5000	1.0000	0.0163 (2)	
O1	0.4123 (6)	0.4986 (4)	0.42179 (18)	0.0220 (9)	
O2	0.2305 (6)	0.2685 (4)	0.37805 (18)	0.0195 (9)	
O3	0.4951 (6)	0.2846 (4)	0.46394 (19)	0.0234 (10)	
O4	0.1821 (6)	0.3677 (4)	0.48335 (17)	0.0175 (9)	
O5	−0.0076 (6)	0.2064 (5)	0.57519 (19)	0.0230 (10)	
O6	−0.3600 (6)	0.1890 (4)	0.59394 (18)	0.0208 (9)	
O7	−0.0972 (7)	0.2861 (5)	0.6784 (2)	0.0366 (12)	
O8	−0.1559 (6)	0.0343 (4)	0.6384 (2)	0.0317 (11)	
O9	0.2770 (6)	0.1287 (4)	0.66017 (17)	0.0152 (8)	
O10	0.2305 (6)	0.0899 (4)	0.75724 (17)	0.0150 (9)	
O11	0.4727 (6)	0.4993 (4)	0.90116 (17)	0.0198 (9)	
O12	0.3876 (7)	0.2637 (4)	0.87580 (18)	0.0261 (10)	
O13	−0.1899 (6)	−0.1202 (4)	0.74975 (17)	0.0210 (9)	
O14	−0.3785 (7)	0.0358 (6)	0.8083 (2)	0.0405 (14)	
O15	−0.3171 (9)	−0.1806 (5)	0.8464 (2)	0.0581 (18)	
O16	−0.0413 (6)	0.0130 (5)	0.84923 (18)	0.0249 (10)	
O17	0.0511 (6)	−0.3579 (4)	0.71192 (17)	0.0190 (9)	
O18	0.2061 (6)	−0.1868 (4)	0.78501 (17)	0.0168 (9)	
O19	0.2704 (7)	−0.1689 (4)	0.91713 (18)	0.0233 (10)	
O20	0.1723 (6)	−0.3106 (4)	0.98679 (18)	0.0208 (9)	
O1W	0.1606 (6)	0.4996 (5)	0.60151 (19)	0.0242 (10)	
O2W	0.2755 (8)	0.0600 (4)	0.5129 (2)	0.0295 (11)	
O3W	0.1524 (7)	−0.1827 (5)	0.57208 (19)	0.0222 (10)	
O4W	0.4233 (6)	−0.1325 (5)	0.6762 (2)	0.0263 (10)	
O5W	−0.2598 (6)	−0.3942 (5)	0.99898 (19)	0.0221 (10)	
O6W	0.1736 (8)	0.0987 (5)	0.9657 (2)	0.0367 (12)	
O7W	0.5970 (7)	0.0679 (5)	0.9443 (2)	0.0269 (10)	
S1	0.3326 (2)	0.35595 (14)	0.43563 (6)	0.0120 (3)	
S2	−0.1535 (2)	0.18259 (15)	0.62287 (6)	0.0136 (3)	
S3	−0.2305 (2)	−0.06454 (16)	0.81416 (7)	0.0186 (3)	
C1	0.4362 (8)	0.3885 (6)	0.8621 (3)	0.0143 (12)	
C2	0.4511 (8)	0.4082 (6)	0.7951 (3)	0.0132 (12)	
C3	0.5229 (9)	0.5306 (6)	0.7176 (3)	0.0181 (13)	
H3	0.5691	0.6064	0.6962	0.022*	
C4	0.3980 (8)	0.3219 (6)	0.7387 (2)	0.0123 (12)	
C5	0.2976 (8)	0.1721 (6)	0.7186 (3)	0.0148 (12)	
C6	0.1110 (8)	−0.3138 (6)	0.7687 (3)	0.0142 (12)	
C7	0.0605 (8)	−0.4112 (6)	0.8153 (3)	0.0133 (12)	
C8	−0.0768 (9)	−0.6127 (6)	0.8472 (3)	0.0161 (13)	
H8	−0.1426	−0.7064	0.8461	0.019*	
C9	0.0754 (8)	−0.3975 (6)	0.8805 (3)	0.0129 (12)	
C10	0.1800 (9)	−0.2830 (6)	0.9302 (3)	0.0169 (13)	
N1	0.4447 (7)	0.4009 (5)	0.6903 (2)	0.0146 (10)	
N2	0.5273 (8)	0.5395 (5)	0.7805 (2)	0.0156 (11)	
N3	−0.0128 (7)	−0.5241 (5)	0.8990 (2)	0.0160 (11)	
N4	−0.0344 (8)	−0.5494 (5)	0.7957 (2)	0.0162 (11)	
H1	−0.054 (9)	−0.587 (6)	0.7570 (12)	0.024*	
H2	0.567 (9)	0.615 (4)	0.808 (2)	0.024*	
H1W	0.069 (6)	0.492 (7)	0.5738 (16)	0.024*	
H2W	0.110 (8)	0.508 (7)	0.6344 (13)	0.024*	
H3W	0.310 (9)	0.047 (5)	0.4774 (15)	0.024*	
H4W	0.233 (9)	−0.022 (3)	0.520 (2)	0.024*	
H5W	0.063 (6)	−0.233 (5)	0.547 (2)	0.024*	
H6W	0.254 (5)	−0.224 (6)	0.565 (3)	0.024*	
H7W	0.498 (7)	−0.116 (7)	0.7088 (13)	0.024*	
H8W	0.500 (7)	−0.112 (7)	0.6495 (17)	0.024*	
H9W	−0.343 (7)	−0.430 (6)	0.9692 (15)	0.024*	
H10W	−0.313 (8)	−0.411 (6)	1.0303 (14)	0.024*	
H11W	0.049 (3)	0.076 (5)	0.958 (3)	0.024*	
H12W	0.187 (8)	0.183 (3)	0.982 (3)	0.024*	
H13W	0.646 (8)	0.148 (3)	0.960 (3)	0.024*	
H14W	0.655 (8)	0.013 (4)	0.962 (3)	0.024*	

### Atomic displacement parameters (Å^2^)

**Table:**

	*U*^11^	*U*^22^	*U*^33^	*U*^12^	*U*^13^	*U*^23^
Gd1	0.01775 (16)	0.01447 (15)	0.01191 (15)	−0.00363 (11)	−0.00195 (12)	0.00366 (12)
Gd2	0.01048 (15)	0.01252 (15)	0.01049 (15)	0.00116 (11)	0.00077 (11)	0.00148 (11)
Gd3	0.01064 (15)	0.01211 (14)	0.01052 (15)	0.00109 (11)	0.00064 (11)	0.00189 (11)
Co1	0.0210 (6)	0.0147 (6)	0.0121 (6)	−0.0023 (5)	0.0020 (5)	0.0029 (5)
O1	0.026 (2)	0.017 (2)	0.020 (2)	−0.0052 (18)	0.0024 (19)	0.0032 (18)
O2	0.018 (2)	0.024 (2)	0.015 (2)	−0.0006 (18)	0.0030 (17)	−0.0005 (18)
O3	0.017 (2)	0.028 (2)	0.027 (2)	0.0068 (19)	−0.0007 (19)	0.011 (2)
O4	0.017 (2)	0.025 (2)	0.010 (2)	0.0011 (17)	0.0034 (17)	0.0016 (17)
O5	0.014 (2)	0.037 (3)	0.021 (2)	0.0037 (19)	0.0061 (18)	0.011 (2)
O6	0.014 (2)	0.027 (2)	0.023 (2)	0.0057 (18)	−0.0016 (18)	0.0068 (19)
O7	0.036 (3)	0.043 (3)	0.026 (3)	0.010 (2)	−0.002 (2)	−0.015 (2)
O8	0.018 (2)	0.017 (2)	0.062 (3)	0.0015 (18)	−0.001 (2)	0.018 (2)
O9	0.015 (2)	0.019 (2)	0.010 (2)	0.0004 (16)	−0.0028 (16)	0.0016 (17)
O10	0.018 (2)	0.013 (2)	0.014 (2)	−0.0029 (16)	0.0035 (17)	0.0071 (17)
O11	0.027 (2)	0.018 (2)	0.012 (2)	0.0025 (18)	−0.0028 (18)	−0.0042 (18)
O12	0.044 (3)	0.020 (2)	0.017 (2)	0.006 (2)	0.009 (2)	0.0068 (19)
O13	0.016 (2)	0.034 (3)	0.010 (2)	−0.0010 (18)	0.0033 (17)	−0.0046 (18)
O14	0.029 (3)	0.079 (4)	0.018 (2)	0.036 (3)	−0.002 (2)	−0.006 (2)
O15	0.094 (5)	0.040 (3)	0.025 (3)	−0.039 (3)	0.018 (3)	−0.006 (2)
O16	0.015 (2)	0.036 (3)	0.020 (2)	0.0000 (19)	0.0022 (18)	−0.006 (2)
O17	0.026 (2)	0.019 (2)	0.011 (2)	0.0022 (18)	0.0000 (18)	0.0020 (17)
O18	0.024 (2)	0.012 (2)	0.015 (2)	0.0008 (17)	0.0042 (18)	0.0034 (17)
O19	0.035 (3)	0.018 (2)	0.015 (2)	−0.0054 (19)	−0.0002 (19)	0.0051 (18)
O20	0.032 (3)	0.015 (2)	0.012 (2)	−0.0080 (18)	−0.0007 (18)	0.0018 (17)
O1W	0.028 (3)	0.030 (2)	0.015 (2)	0.011 (2)	0.0010 (19)	0.002 (2)
O2W	0.045 (3)	0.015 (2)	0.023 (2)	−0.010 (2)	0.007 (2)	−0.0016 (19)
O3W	0.017 (2)	0.026 (2)	0.021 (2)	0.0026 (19)	−0.0025 (19)	−0.0031 (19)
O4W	0.016 (2)	0.043 (3)	0.021 (2)	0.010 (2)	0.0017 (19)	0.002 (2)
O5W	0.024 (3)	0.027 (2)	0.013 (2)	−0.0004 (19)	0.0008 (19)	−0.001 (2)
O6W	0.045 (3)	0.028 (3)	0.034 (3)	0.006 (2)	0.002 (3)	−0.005 (2)
O7W	0.032 (3)	0.022 (2)	0.022 (2)	−0.001 (2)	−0.012 (2)	0.001 (2)
S1	0.0114 (7)	0.0136 (7)	0.0105 (7)	−0.0011 (5)	0.0005 (6)	0.0031 (6)
S2	0.0117 (7)	0.0140 (7)	0.0151 (7)	0.0024 (6)	0.0006 (6)	0.0018 (6)
S3	0.0143 (8)	0.0242 (8)	0.0148 (8)	−0.0008 (6)	0.0030 (6)	−0.0036 (6)
C1	0.011 (3)	0.018 (3)	0.013 (3)	0.004 (2)	−0.004 (2)	−0.001 (3)
C2	0.012 (3)	0.010 (3)	0.017 (3)	0.002 (2)	0.000 (2)	0.000 (2)
C3	0.025 (3)	0.014 (3)	0.013 (3)	−0.001 (3)	0.003 (3)	0.002 (2)
C4	0.011 (3)	0.017 (3)	0.009 (3)	0.000 (2)	−0.001 (2)	0.004 (2)
C5	0.009 (3)	0.017 (3)	0.017 (3)	0.000 (2)	−0.002 (2)	−0.002 (3)
C6	0.013 (3)	0.019 (3)	0.012 (3)	0.007 (2)	0.004 (2)	0.002 (2)
C7	0.011 (3)	0.016 (3)	0.013 (3)	0.003 (2)	−0.001 (2)	0.002 (2)
C8	0.016 (3)	0.014 (3)	0.019 (3)	0.001 (2)	0.003 (3)	0.005 (3)
C9	0.011 (3)	0.012 (3)	0.015 (3)	0.002 (2)	0.002 (2)	0.004 (2)
C10	0.014 (3)	0.018 (3)	0.019 (3)	0.006 (3)	0.002 (3)	0.001 (3)
N1	0.015 (3)	0.015 (3)	0.013 (2)	0.001 (2)	−0.001 (2)	0.002 (2)
N2	0.023 (3)	0.012 (3)	0.010 (3)	−0.001 (2)	0.003 (2)	−0.001 (2)
N3	0.020 (3)	0.014 (2)	0.013 (3)	−0.001 (2)	0.003 (2)	0.003 (2)
N4	0.024 (3)	0.014 (3)	0.010 (2)	0.000 (2)	0.002 (2)	0.000 (2)

### Geometric parameters (Å, °)

**Table:**

Gd1—O19	2.276 (4)	O9—C5	1.266 (6)
Gd1—O12	2.277 (4)	O10—C5	1.270 (6)
Gd1—O14^i^	2.364 (5)	O11—C1	1.245 (6)
Gd1—O16	2.381 (4)	O12—C1	1.259 (7)
Gd1—O18	2.382 (4)	O13—S3	1.480 (4)
Gd1—O7W	2.419 (4)	O14—S3	1.469 (4)
Gd1—O10	2.462 (4)	O14—Gd1^v^	2.364 (4)
Gd1—O6W	2.593 (5)	O15—S3	1.446 (5)
Gd2—O8	2.341 (4)	O16—S3	1.463 (4)
Gd2—O13	2.369 (4)	O17—C6	1.256 (7)
Gd2—O2^ii^	2.396 (4)	O18—C6	1.273 (7)
Gd2—O4W	2.423 (4)	O19—C10	1.235 (7)
Gd2—O3W	2.423 (4)	O20—C10	1.281 (7)
Gd2—O10	2.496 (4)	O1W—H1W	0.81 (2)
Gd2—O17	2.504 (4)	O1W—H2W	0.81 (4)
Gd2—O9	2.616 (4)	O2W—H3W	0.81 (4)
Gd2—O18	2.641 (4)	O2W—H4W	0.83 (2)
Gd2—C6	2.919 (5)	O3W—H5W	0.83 (2)
Gd2—C5	2.934 (6)	O3W—H6W	0.84 (4)
Gd3—O6^i^	2.314 (4)	O4W—H7W	0.81 (2)
Gd3—O1^iii^	2.357 (4)	O4W—H8W	0.82 (5)
Gd3—O5	2.374 (4)	O5W—H9W	0.82 (2)
Gd3—O2W	2.428 (4)	O5W—H10W	0.81 (4)
Gd3—O4	2.466 (4)	O6W—H11W	0.82 (2)
Gd3—O1W	2.490 (4)	O6W—H12W	0.82 (2)
Gd3—N1	2.510 (4)	O7W—H13W	0.80 (2)
Gd3—O9	2.525 (4)	O7W—H14W	0.81 (5)
Gd3—O3	2.703 (4)	C1—C2	1.487 (7)
Gd3—S1	3.1872 (13)	C2—N2	1.360 (7)
Co1—O20^iv^	2.051 (4)	C2—C4	1.369 (8)
Co1—O20	2.051 (4)	C3—N1	1.313 (7)
Co1—O5W	2.112 (4)	C3—N2	1.343 (7)
Co1—O5W^iv^	2.112 (4)	C3—H3	0.9300
Co1—N3	2.151 (4)	C4—N1	1.385 (7)
Co1—N3^iv^	2.151 (4)	C4—C5	1.479 (8)
O1—S1	1.456 (4)	C6—C7	1.471 (7)
O1—Gd3^iii^	2.357 (4)	C7—N4	1.372 (7)
O2—S1	1.464 (4)	C7—C9	1.389 (7)
O2—Gd2^ii^	2.396 (4)	C8—N3	1.315 (7)
O3—S1	1.482 (4)	C8—N4	1.352 (7)
O4—S1	1.490 (4)	C8—H8	0.9300
O5—S2	1.478 (4)	C9—N3	1.377 (7)
O6—S2	1.472 (4)	C9—C10	1.481 (8)
O6—Gd3^v^	2.314 (4)	N2—H2	0.87 (2)
O7—S2	1.440 (5)	N4—H1	0.86 (2)
O8—S2	1.484 (4)		
			
O19—Gd1—O12	140.28 (14)	O20^iv^—Co1—O5W	91.67 (16)
O19—Gd1—O14^i^	115.09 (16)	O20—Co1—O5W	88.33 (16)
O12—Gd1—O14^i^	84.34 (17)	O20^iv^—Co1—O5W^iv^	88.33 (16)
O19—Gd1—O16	88.40 (15)	O20—Co1—O5W^iv^	91.67 (16)
O12—Gd1—O16	93.73 (15)	O5W—Co1—O5W^iv^	180.000 (3)
O14^i^—Gd1—O16	144.89 (14)	O20^iv^—Co1—N3	100.80 (16)
O19—Gd1—O18	74.59 (13)	O20—Co1—N3	79.20 (16)
O12—Gd1—O18	144.34 (13)	O5W—Co1—N3	91.23 (16)
O14^i^—Gd1—O18	84.41 (16)	O5W^iv^—Co1—N3	88.77 (16)
O16—Gd1—O18	76.91 (14)	O20^iv^—Co1—N3^iv^	79.20 (16)
O19—Gd1—O7W	74.98 (15)	O20—Co1—N3^iv^	100.80 (16)
O12—Gd1—O7W	77.45 (15)	O5W—Co1—N3^iv^	88.77 (16)
O14^i^—Gd1—O7W	75.42 (16)	O5W^iv^—Co1—N3^iv^	91.23 (16)
O16—Gd1—O7W	138.43 (15)	N3—Co1—N3^iv^	180.000 (3)
O18—Gd1—O7W	131.41 (14)	S1—O1—Gd3^iii^	157.6 (3)
O19—Gd1—O10	141.86 (14)	S1—O2—Gd2^ii^	150.4 (2)
O12—Gd1—O10	75.24 (13)	S1—O3—Gd3	94.70 (19)
O14^i^—Gd1—O10	73.07 (13)	S1—O4—Gd3	104.62 (19)
O16—Gd1—O10	72.56 (13)	S2—O5—Gd3	135.3 (2)
O18—Gd1—O10	69.11 (12)	S2—O6—Gd3^v^	154.8 (3)
O7W—Gd1—O10	139.82 (14)	S2—O8—Gd2	141.0 (3)
O19—Gd1—O6W	70.00 (15)	C5—O9—Gd3	123.4 (3)
O12—Gd1—O6W	74.41 (15)	C5—O9—Gd2	91.4 (3)
O14^i^—Gd1—O6W	143.57 (17)	Gd3—O9—Gd2	142.26 (16)
O16—Gd1—O6W	67.18 (15)	C5—O10—Gd1	141.8 (3)
O18—Gd1—O6W	129.32 (15)	C5—O10—Gd2	96.9 (3)
O7W—Gd1—O6W	71.34 (16)	Gd1—O10—Gd2	113.81 (13)
O10—Gd1—O6W	126.92 (14)	C1—O12—Gd1	157.9 (4)
O8—Gd2—O13	78.04 (15)	S3—O13—Gd2	143.5 (2)
O8—Gd2—O2^ii^	73.04 (14)	S3—O14—Gd1^v^	122.0 (3)
O13—Gd2—O2^ii^	75.19 (13)	S3—O16—Gd1	143.1 (2)
O8—Gd2—O4W	135.66 (15)	C6—O17—Gd2	96.2 (3)
O13—Gd2—O4W	138.59 (14)	C6—O18—Gd1	152.1 (3)
O2^ii^—Gd2—O4W	129.93 (14)	C6—O18—Gd2	89.4 (3)
O8—Gd2—O3W	90.09 (15)	Gd1—O18—Gd2	111.49 (14)
O13—Gd2—O3W	148.44 (14)	C10—O19—Gd1	155.2 (4)
O2^ii^—Gd2—O3W	73.39 (14)	C10—O20—Co1	117.3 (4)
O4W—Gd2—O3W	68.07 (14)	Gd3—O1W—H1W	107 (4)
O8—Gd2—O10	89.77 (14)	Gd3—O1W—H2W	118 (4)
O13—Gd2—O10	81.20 (13)	H1W—O1W—H2W	107 (3)
O2^ii^—Gd2—O10	153.11 (13)	Gd3—O2W—H3W	123 (4)
O4W—Gd2—O10	76.75 (14)	Gd3—O2W—H4W	133 (4)
O3W—Gd2—O10	128.41 (13)	H3W—O2W—H4W	103 (3)
O8—Gd2—O17	140.55 (14)	Gd2—O3W—H5W	117 (4)
O13—Gd2—O17	76.30 (13)	Gd2—O3W—H6W	123 (4)
O2^ii^—Gd2—O17	71.75 (13)	H5W—O3W—H6W	101 (3)
O4W—Gd2—O17	81.93 (15)	Gd2—O4W—H7W	120 (4)
O3W—Gd2—O17	96.39 (13)	Gd2—O4W—H8W	129 (4)
O10—Gd2—O17	115.04 (12)	H7W—O4W—H8W	104 (3)
O8—Gd2—O9	70.37 (13)	Co1—O5W—H9W	111 (4)
O13—Gd2—O9	120.85 (13)	Co1—O5W—H10W	105 (4)
O2^ii^—Gd2—O9	134.71 (12)	H9W—O5W—H10W	106 (3)
O4W—Gd2—O9	68.39 (14)	Gd1—O6W—H11W	104 (4)
O3W—Gd2—O9	80.86 (13)	Gd1—O6W—H12W	122 (4)
O10—Gd2—O9	50.97 (11)	H11W—O6W—H12W	104 (3)
O17—Gd2—O9	149.08 (12)	Gd1—O7W—H13W	123 (4)
O8—Gd2—O18	140.60 (14)	Gd1—O7W—H14W	129 (4)
O13—Gd2—O18	69.20 (13)	H13W—O7W—H14W	108 (3)
O2^ii^—Gd2—O18	116.86 (12)	O1—S1—O2	109.4 (2)
O4W—Gd2—O18	69.74 (13)	O1—S1—O3	112.1 (3)
O3W—Gd2—O18	129.17 (13)	O2—S1—O3	110.7 (2)
O10—Gd2—O18	64.61 (12)	O1—S1—O4	109.6 (2)
O17—Gd2—O18	50.43 (12)	O2—S1—O4	109.2 (2)
O9—Gd2—O18	108.33 (12)	O3—S1—O4	105.7 (2)
O8—Gd2—C6	144.12 (15)	O1—S1—Gd3	119.68 (17)
O13—Gd2—C6	66.49 (14)	O2—S1—Gd3	130.33 (17)
O2^ii^—Gd2—C6	92.18 (15)	O3—S1—Gd3	57.69 (16)
O4W—Gd2—C6	78.83 (15)	O4—S1—Gd3	48.48 (15)
O3W—Gd2—C6	117.36 (14)	O7—S2—O6	112.2 (3)
O10—Gd2—C6	90.03 (14)	O7—S2—O5	110.9 (3)
O17—Gd2—C6	25.32 (14)	O6—S2—O5	107.9 (2)
O9—Gd2—C6	132.97 (14)	O7—S2—O8	110.7 (3)
O18—Gd2—C6	25.85 (14)	O6—S2—O8	106.5 (2)
O8—Gd2—C5	78.12 (15)	O5—S2—O8	108.5 (3)
O13—Gd2—C5	100.76 (15)	O15—S3—O16	110.6 (3)
O2^ii^—Gd2—C5	151.11 (14)	O15—S3—O14	109.3 (3)
O4W—Gd2—C5	71.80 (16)	O16—S3—O14	109.0 (3)
O3W—Gd2—C5	105.28 (15)	O15—S3—O13	110.6 (3)
O10—Gd2—C5	25.46 (13)	O16—S3—O13	110.0 (2)
O17—Gd2—C5	135.95 (14)	O14—S3—O13	107.3 (2)
O9—Gd2—C5	25.55 (13)	O11—C1—O12	124.5 (5)
O18—Gd2—C5	86.90 (14)	O11—C1—C2	116.5 (5)
C6—Gd2—C5	112.75 (16)	O12—C1—C2	118.9 (5)
O6^i^—Gd3—O1^iii^	82.80 (14)	N2—C2—C4	105.7 (5)
O6^i^—Gd3—O5	133.45 (14)	N2—C2—C1	119.3 (5)
O1^iii^—Gd3—O5	143.43 (14)	C4—C2—C1	134.9 (5)
O6^i^—Gd3—O2W	75.16 (16)	N1—C3—N2	111.2 (5)
O1^iii^—Gd3—O2W	134.40 (14)	N1—C3—H3	124.4
O5—Gd3—O2W	71.70 (16)	N2—C3—H3	124.4
O6^i^—Gd3—O4	132.10 (13)	C2—C4—N1	109.2 (5)
O1^iii^—Gd3—O4	84.69 (13)	C2—C4—C5	135.6 (5)
O5—Gd3—O4	73.56 (13)	N1—C4—C5	115.2 (5)
O2W—Gd3—O4	81.54 (14)	O9—C5—O10	120.6 (5)
O6^i^—Gd3—O1W	148.84 (15)	O9—C5—C4	116.8 (5)
O1^iii^—Gd3—O1W	73.71 (15)	O10—C5—C4	122.6 (5)
O5—Gd3—O1W	70.77 (15)	O9—C5—Gd2	63.1 (3)
O2W—Gd3—O1W	136.00 (16)	O10—C5—Gd2	57.7 (3)
O4—Gd3—O1W	66.48 (13)	C4—C5—Gd2	174.7 (4)
O6^i^—Gd3—N1	82.54 (14)	O17—C6—O18	120.5 (5)
O1^iii^—Gd3—N1	72.44 (14)	O17—C6—C7	118.0 (5)
O5—Gd3—N1	103.43 (14)	O18—C6—C7	121.5 (5)
O2W—Gd3—N1	140.51 (15)	O17—C6—Gd2	58.5 (3)
O4—Gd3—N1	136.00 (13)	O18—C6—Gd2	64.8 (3)
O1W—Gd3—N1	71.16 (14)	C7—C6—Gd2	160.3 (4)
O6^i^—Gd3—O9	73.82 (13)	N4—C7—C9	104.9 (5)
O1^iii^—Gd3—O9	131.86 (13)	N4—C7—C6	119.9 (5)
O5—Gd3—O9	68.63 (13)	C9—C7—C6	135.1 (5)
O2W—Gd3—O9	79.16 (13)	N3—C8—N4	111.0 (5)
O4—Gd3—O9	141.29 (13)	N3—C8—H8	124.5
O1W—Gd3—O9	107.16 (13)	N4—C8—H8	124.5
N1—Gd3—O9	63.36 (13)	N3—C9—C7	109.3 (5)
O6^i^—Gd3—O3	77.88 (12)	N3—C9—C10	117.6 (5)
O1^iii^—Gd3—O3	71.57 (13)	C7—C9—C10	132.8 (5)
O5—Gd3—O3	114.80 (13)	O19—C10—O20	122.7 (6)
O2W—Gd3—O3	65.09 (14)	O19—C10—C9	121.5 (5)
O4—Gd3—O3	54.32 (12)	O20—C10—C9	115.9 (5)
O1W—Gd3—O3	112.45 (13)	C3—N1—C4	105.7 (4)
N1—Gd3—O3	140.72 (14)	C3—N1—Gd3	133.5 (4)
O9—Gd3—O3	138.93 (12)	C4—N1—Gd3	120.5 (3)
O6^i^—Gd3—S1	105.48 (10)	C3—N2—C2	108.2 (5)
O1^iii^—Gd3—S1	74.47 (10)	C3—N2—H2	128 (4)
O5—Gd3—S1	95.66 (10)	C2—N2—H2	124 (4)
O2W—Gd3—S1	73.79 (11)	C8—N3—C9	106.4 (5)
O4—Gd3—S1	26.90 (9)	C8—N3—Co1	144.3 (4)
O1W—Gd3—S1	87.89 (10)	C9—N3—Co1	109.3 (4)
N1—Gd3—S1	144.61 (11)	C8—N4—C7	108.3 (5)
O9—Gd3—S1	152.00 (9)	C8—N4—H1	128 (4)
O3—Gd3—S1	27.62 (8)	C7—N4—H1	123 (4)
O20^iv^—Co1—O20	180.00 (13)		

Symmetry codes: (i) *x*+1, *y*, *z*; (ii) −*x*, −*y*, −*z*+1; (iii) −*x*+1, −*y*+1, −*z*+1; (iv) −*x*, −*y*−1, −*z*+2; (v) *x*−1, *y*, *z*.

### Hydrogen-bond geometry (Å, °)

**Table:**

*D*—H···*A*	*D*—H	H···*A*	*D*···*A*	*D*—H···*A*
N4—H1···O7^vi^	0.86 (3)	1.93 (4)	2.770 (6)	166 (5)
N2—H2···O15^vii^	0.87 (4)	2.03 (4)	2.865 (7)	161 (4)
O1W—H2W···O17^viii^	0.81 (4)	2.07 (4)	2.785 (6)	148 (6)
O2W—H4W···O3W	0.83 (3)	2.04 (4)	2.817 (6)	158 (4)
O3W—H5W···O4^ii^	0.82 (4)	1.93 (4)	2.720 (6)	159 (4)
O3W—H6W···O3^ix^	0.84 (4)	1.96 (4)	2.779 (6)	167 (6)
O4W—H7W···O13^i^	0.81 (4)	2.19 (5)	2.888 (6)	144 (4)
O4W—H7W···O14^i^	0.81 (4)	2.44 (4)	3.175 (6)	150 (5)
O4W—H8W···O8^i^	0.82 (5)	2.54 (5)	3.217 (6)	141 (4)
O4W—H8W···O2^ix^	0.82 (5)	2.54 (5)	3.028 (6)	120 (5)
O5W—H9W···O11^x^	0.82 (4)	1.83 (4)	2.645 (6)	177 (6)
O5W—H10W···O11^xi^	0.81 (4)	2.07 (5)	2.862 (6)	167 (5)
O5W—H10W···O12^xi^	0.81 (4)	2.44 (4)	3.056 (6)	134 (5)
O6W—H12W···O5W^xi^	0.82 (4)	1.98 (3)	2.779 (7)	164 (6)
O7W—H13W···O20^xii^	0.81 (4)	1.99 (5)	2.768 (6)	161 (4)
O7W—H14W···O6W^xii^	0.81 (5)	2.31 (5)	3.090 (7)	165 (5)
C3—H3···O2^iii^	0.93	2.46	3.336 (7)	157

Symmetry codes: (vi) *x*, *y*−1, *z*; (vii) *x*+1, *y*+1, *z*; (viii) *x*, *y*+1, *z*; (ii) −*x*, −*y*, −*z*+1; (ix) −*x*+1, −*y*, −*z*+1; (i) *x*+1, *y*, *z*; (x) *x*−1, *y*−1, *z*; (xi) −*x*, −*y*, −*z*+2; (xii) −*x*+1, −*y*, −*z*+2; (iii) −*x*+1, −*y*+1, −*z*+1.
